# Diel Vertical Migration and Transport Pattern of Larvae and Juveniles of the Small Yellow Croaker (*Larimichthys polyactis*) in the Yangtze River Estuary

**DOI:** 10.3390/ani15081128

**Published:** 2025-04-14

**Authors:** Xiaojing Song, Fen Hu, Jianzhong Ling, Xingwei Yuan, Zunlei Liu, Yan Jin, Shengfa Li, Yazhou Jiang

**Affiliations:** 1 East China Sea Fisheries Research Institute, Chinese Academy of Fishery Sciences, Shanghai 200090, China; songxiaojing@ecsf.ac.cn (X.S.); huf@ecsf.ac.cn (F.H.); yuanxw@ecsf.ac.cn (X.Y.); liuzl@ecsf.ac.cn (Z.L.); jiny@ecsf.ac.cn (Y.J.); lisf@ecsf.ac.cn (S.L.); 2 Key Laboratory of East China Sea Fishery Resources Exploitation and Utilization, Ministry of Agriculture and Rural Affairs, Shanghai 200090, China

**Keywords:** small yellow croaker, larvae and juveniles, vertical distribution, transport pattern, Yangtze River estuary

## Abstract

In order to understand the diel vertical migration and transport pattern during their early life stages, the vertical distribution of larvae and juveniles of the small yellow croaker (*Larimichthys polyactis*) in the Yangtze River estuary was investigated. Four sampling cycles were carried out at the station (122°39′ E, 31° N) during May and June 2015. The samples were collected by vertically discrete plankton hauls in three strata of the water column: surface (0~2 m depth), middle (8~10 m depth), and bottom (18~20 m depth). The total number of small yellow croaker larvae was 5523, of which 65.2% and 25.8% were postflexion larvae and juveniles, respectively. Most of the individuals were collected in mid-May, and the sum of the quantity in May accounted for 94.8%. In all cruises, the highest abundance occurred at the bottom, while the surface maintained the lowest abundance. The flexion larvae, postflexion larvae and juveniles moved up to the surface and middle at night, but there was no significant difference between night and daytime for preflexion larvae in each stratum. The postflexion larvae and juveniles showed low temperature responses and a preference for the bottom habitat, respectively, while the preflexion and flexion larvae had weaker selectivity to the water layer and occurrence time. The abundance of larvae and juveniles in the middle layer was significantly higher at flood tide than at ebb tide, and it was more dominant at night than during the day. The study indicates that larvae and juveniles of the small yellow croaker change their located depth with diel vertical migration and utilize the faster speed of the mid-upper layer, which contributes to their ingress into the Yangtze River estuary nursery ground.

## Simple Summary

For successful lifecycle completion, some fish species must experience an inshore migration stage, gradually migrating from the open sea to the surf area to find a more suitable habitat during their early life stages. The Yangtze River estuary has always been an important nursery ground for the small yellow croaker, although recruitment into the estuarine nursery area is challenging, as there is a net flow of water from the estuary to the ocean and current speeds often exceed larval swimming speeds. Larvae and juveniles of the small yellow croaker recruiting into the estuary must therefore adopt strategies for successful ingress into the estuarine nursery ground. For this reason, a fixed station was used to examine the vertical distribution and explore the transport pattern of the larval small yellow croaker in the Yangtze River estuary. During every cruise, the highest and lowest abundances occurred at the bottom and on the surface, respectively. The larval small yellow croaker moved up to the surface and middle at night, with its abundance varying with periodic currents. The findings indicate that the larvae move up to the water strata in which the current speed is faster during flood tides and move down to the deeper water in which the current speed is slower during ebb tides. Depending on this pattern, the larvae and juveniles of the small yellow croaker can ingress into the Yangtze River estuary nursery ground.

## 1. Introduction

For many marine fish species, recruitment to the adult population requires transportation from open ocean spawning regions to estuarine nursery habitats during their early life stages [1,2]. However, there is a net flow of water to the ocean and the current speed frequently exceeds the larval swimming speed [3,4,5]. Consequently, fish larvae must adopt appropriate strategies for successful ingress into estuarine grounds. They not only drift passively in the current, but also migrate vertically to actively selective preference depths with different stratified current speeds and then move toward the estuarine nursery grounds [6].

Vertical migration has been recognized as a critical component of realistic models of larval fish dispersion [7]. In general, fish larvae have heterogeneous vertical distribution [8], and display species-specific vertical distribution [9,10]. Larvae of many fish species migrate vertically, on a diel basis [11] or ontogenetically [6]. The study of vertical distribution patterns of fish larvae is essential to obtaining a better understanding of their ecology [12] because feeding, predation and larval transport vary considerably with depth [13]. Also, knowledge of the vertical distribution of fish larvae has practical implications for sampling designs [14], which can have a strong influence on the interpretation of results from ichthyoplankton surveys [15].

The small yellow croaker (*Larimichthys polyactis*) is a commercially important fish that is widely distributed in the estuaries and coastal waters of the East China Sea, Yellow Sea and Bohai Sea [16]. It migrates toward the coastal waters of China from overwintering grounds for spawning in the spring [17]. In the southern part of the Yellow Sea, the spawning time of the small yellow croaker is usually from April to May, and the main spawning grounds include the Haizhou Bay spawning ground and the Lüsi spawning ground. In the East China Sea, the spawning time of the small yellow croaker is from March to May, and the main spawning grounds are located in the Yangtze River estuary (YRE) and sea areas, such as Dongting, Jiushan, Yushan, Dachen and Dongtoushan, along the coast of Zhejiang [18]. Since before the 1980s, the YRE has always been an important spawning and nursery ground for the small yellow croaker [19]. However, recent studies have shown that the small yellow croaker mainly spawns in the outer waters and only enters the YRE to use it as a nursery ground [20,21,22].

The YRE is the largest estuary in China. It connects rivers and seas and has unique hydrology and water quality conditions. The huge runoff of the Yangtze River brings a large amount of silt to the YRE, forming a unique landscape ecosystem that has become an important habitat for fish. The Yangtze River runoff also transports a large amount of nutrients to the YRE, making it an important place for the spawning, foraging and breeding of various economic fish [23]. The distinct hydrodynamic and tidal currents may play an important role in the transport and migratory potential during the early life stages of fish, especially for pelagic larvae with ontogenetic variations in swimming abilities [24].

The importance of dispersal and transport for fish larvae is indubitable because the physical and biological processes that promote the aggregation of larvae in appropriate conditions possibly determine their survival [25]. The connectivity between spawning and nursery areas may be one of the major determinants of the dynamics of fish populations [26]. An increasing number of studies indicate that the spawning ground of the small yellow croaker is located outside the YRE, and larvae need to migrate from the open sea to the surf area to find a more suitable habitat during their early life stages. Due to the complex habitat of the YRE and the influence of the Yangtze River runoff, it is very difficult for the small yellow croaker to successfully migrate to the nursery ground of the YRE. How the larvae and juveniles of the small yellow croaker migrate from the offshore spawning ground to the coastal nursery ground of the YRE needs to be intensively studied. Therefore, the objectives of this study are to examine the vertical distribution and explore the transport pattern of larvae and juveniles of the small yellow croaker in the YRE.

## 2. Materials and Methods

### 2.1. Sampling

**Figure 1:**
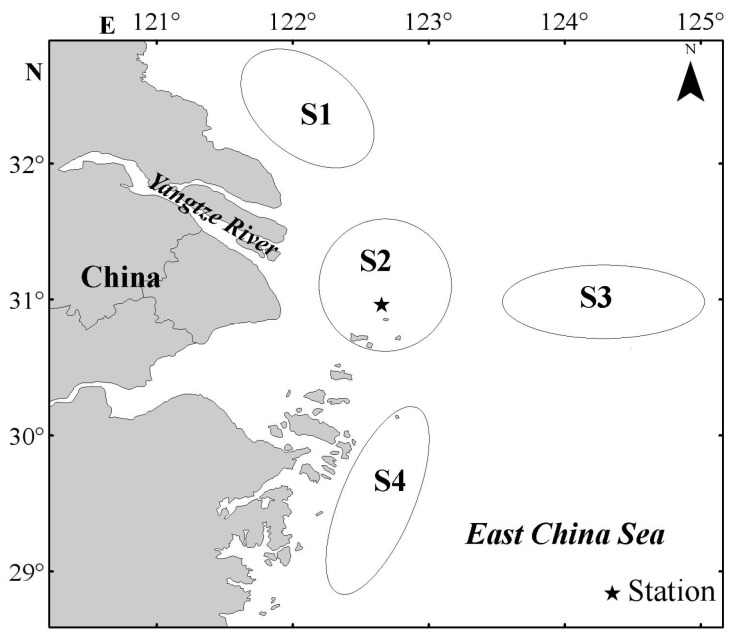
Sampling station and schematic diagram of the spawning grounds of the small yellow croaker. S1: Lüsi spawning ground; S2: Sheshan Island spawning ground; S3: Outer Yangtze River estuary spawning ground; S4: Zhejiang coastal spawning ground.

The sampling was carried out in early May (5 May), mid-May (20 May), early June (3 June) and mid-June (18 June) in 2015 at a station (122°39′ E, 31° N) in the YRE (Figure 1). Ichthyoplankton were collected by conical–cylindrical plankton nets (1.3 m mouth diameter, 6 m length and 0.5 mm mesh), and each net was equipped with a flowmeter to estimate the volume of filtered water. Three strata in the water column were sampled: surface (0~2 m depth), middle (8~10 m depth), and bottom (18~20 m depth), depending on tidal conditions. Sampling depth was determined from the length of the towrope and angle (45°), and the top of the net was just below the surface layer [27]. Plankton hauls were taken continuously for 24 h during every sampling cycle, which commenced at 07:00 and performed at regular intervals of 3 h. A total of 8 hauls were taken and 24 samples were collected from three water layers during each cruise, so a total of 96 samples were obtained from 4 cruises. In each haul, the net was passively fished for 10 min against the tidal flow. Meanwhile, the vertical temperature and salinity were taken with an SBE 19plus V2 SEACAT profiler (Sea-Bird Electronics, Inc., Bellevue, WA, USA), and the current speed and direction were taken with a FlowQuest 600 acoustic current profiler (LinkQuest, Inc., San Diego, CA, USA).

### 2.2. Data Analysis

The ichthyoplankton samples were fixed with 5% seawater formalin immediately after collection. In the laboratory, the samples were sorted, counted and measured (mm, standard length (SL)) under a stereomicroscope. Fish were identified to the lowest possible taxonomic level using morphometric and meristic characteristics [28]. The primary focus of our study was the larvae and juveniles of the small yellow croaker. The larvae are divided into four stages, including yolk-sac, preflexion, flexion and postflexion; the juvenile stage follows the larval stage, beginning when fin ray counts are complete and ending with the completion of squamation development. The variations in hydrographic conditions and the abundance of the larvae and juveniles were analyzed by factorial analysis of variance (ANOVA). All variables were checked for normality and homogeneity of variance using Shapiro–Wilk and Levene tests, respectively and, where necessary, log (X + 1) transformed before further statistical analysis. A post hoc comparison was performed with the Tukey HSD method if any significance was found during the ANOVA.

Redundancy analysis (RDA) was used to parse the driving mechanism of environmental factors on the distribution pattern of each developmental stage of the larvae and juveniles of the small yellow croaker. RDA, as a constrained linear ordination method, quantified the explanatory role of the environmental factor matrix on the species distribution matrix by establishing a multiple model [29]. Data analysis followed the following process: first, standardize the species data and transform the density of larvae and juveniles at each developmental stage by Hellinger [30] to eliminate the effect of excessive zero value on the calculation of Euclidean distance; second, implement Z-score standardization for environmental variables to eliminate dimensional differences. Collinearity was assessed by the variance inflation factor (VIF), with all variables having VIF values below the threshold of 10 (maximum VIF = 2.5), indicating that high collinearity variables did not need to be removed. Subsequently, the forward selection method, combined with the Monte Carlo permutation test (999 permutations, α = 0.05), was used to screen for significant environmental variables [31], ultimately retaining three key factors: water temperature, sampling water layer and sampling time (day/night). The constraint model was constructed based on the rda () function of the vegan software package v2.6-10 [32], with the filtered environmental variables as the explanatory matrix. The overall significance of the model was evaluated by a permutation test with 999 replicates, and the adjusted coefficient of determination (R^2^adj) was calculated to quantify the proportion of explained variance. The eigenvalue decomposition method was used for the ecological significance of the sorting axis, and the cumulative interpretation rate of the first two axes represented the core environmental gradient. The result visualization was implemented in the ggplot2 package v3.4.2 [33]. The constructed three-order diagram synchronously displayed the sample square distribution, the species response vector and the direction of the environmental factor constraints.

The relationship between fish abundance and current velocity and direction was presented by GeoGebra [34]. According to the direction of the tidal current, this study stipulates that the larvae and juveniles move toward the shore (flood tide) between 180° and 360° and offshore (ebb tide) between 0° and 180°.

## 3. Results

### 3.1. Hydrographic Conditions

**Figure 2:**
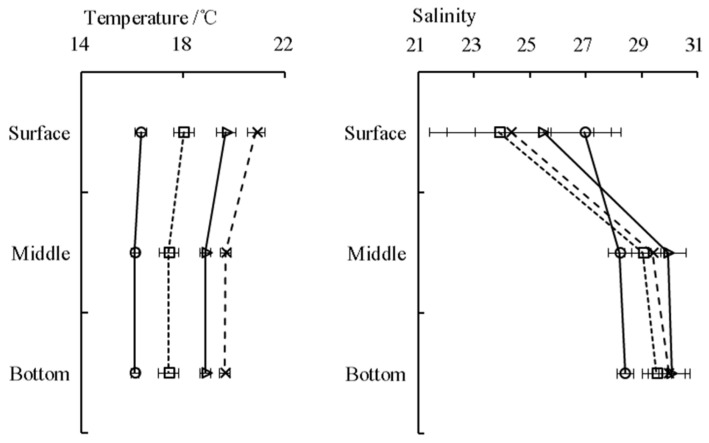
Temperature (°C) and salinity during early May (○), mid-May (□), early June (△), and mid-June (×).

**Figure 3:**
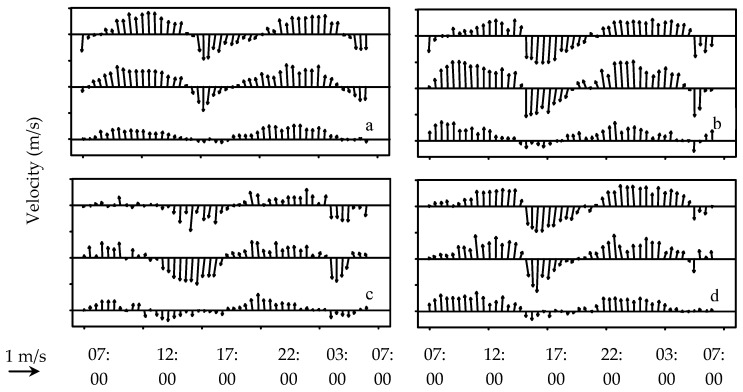
Observed velocity and direction of current during early May (**a**), mid-May (**b**), early June (**c**), and mid-June (**d**). Data temporal and spatial resolutions are 0.5 h and 5 m, respectively.

No significant temperature difference was observed among the three depth strata (*p* > 0.05) in every sampling cycle, while the surface temperature was slightly higher (Figure 2). The values of surface salinity were lower than the other two depth strata and fluctuated acutely during every sampling cycle (Figure 2). The observed velocity and direction of current during four sampling cycles indicated that the tide pattern belongs to the regular semi-diurnal tides in the YRE (Figure 3). The changes in current direction in the three depth strata occurred simultaneously during every sampling cycle, indicating that there was no obvious vertical stratification in the YRE. However, the speeds measured in the surface and middle water, with average values of 0.80 m/s and 0.79 m/s respectively, were significantly faster (*p* < 0.05) than those in the bottom layer (0.36 m/s).

### 3.2. Abundance

**Figure 4:**
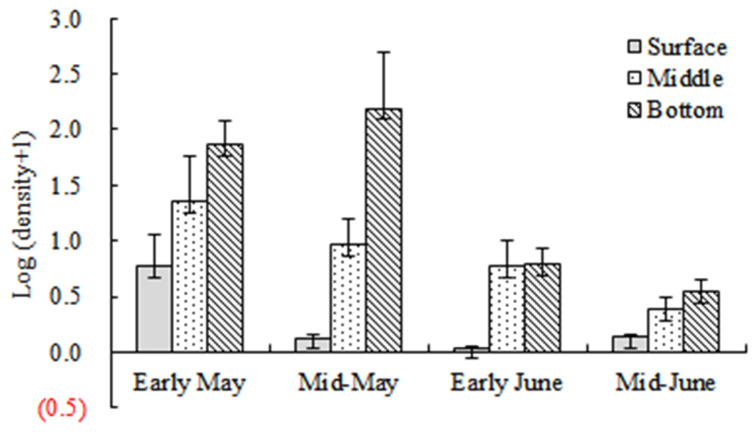
The logarithm values of abundance in the three depth strata during four sampling cycles.

A total of 5523 larvae and juveniles were collected. The results of factorial ANOVA showed that month and water layer had significant effects on the abundance of the larvae and juveniles (F = 25.30, *p* < 0.05; F = 49.37, *p* < 0.05) (Table 1). 35.6% and 59.2% of the total individuals were collected in early May and mid-May, respectively, which were significantly higher than those collected in early June and mid-June (*p* < 0.05). Through all the investigations, the highest abundance (average abundance 58.3 ind./100 m^3^) occurred in the bottom stratum, while the surface maintained the lowest abundance (average abundance 1.4 ind./100 m^3^) (Table 2). Significant differences were seen among the three depth strata (*p* < 0.05), except at the middle and the bottom in early June (Figure 4).

**Figure 5:**
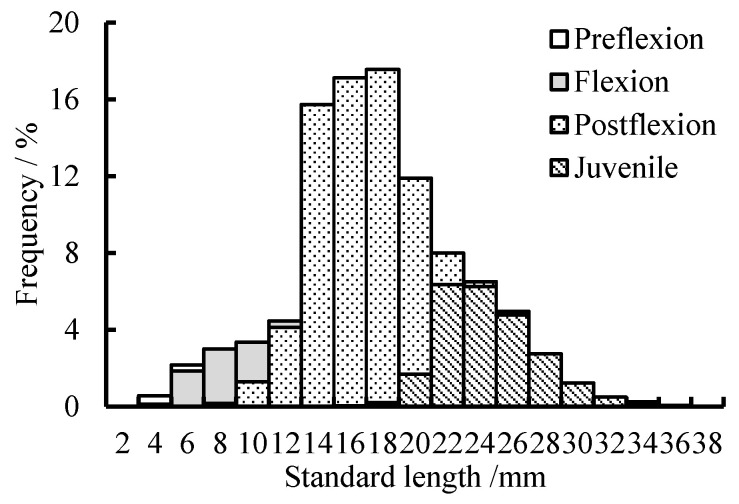
The length–frequency distribution of individuals at different development stages.

The standard length (SL) of all specimens ranged from 4.5 to 37.6 mm (Figure 5). The mean SL was (5.8 ± 0.7) mm for preflexion larvae, (9.4 ± 1.6) mm for flexion larvae, (17.5 ± 3.2) mm for postflexion larvae and (25.6 ± 3.2) mm for juveniles. Postflexion larvae and juveniles dominated the total catches, accounting for 65.2% and 25.8%, respectively; preflexion and flexion larvae represented only 0.5% and 8.5%, respectively.

### 3.3. Diel Vertical Distribution

**Figure 6:**
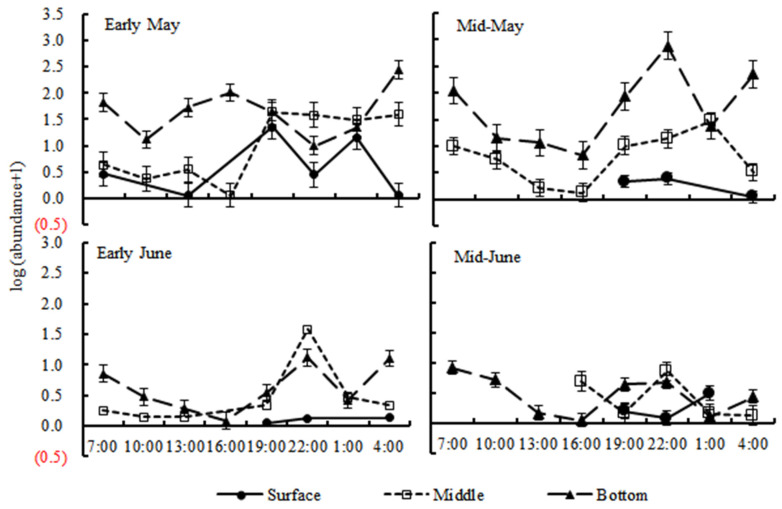
The logarithmic value of abundance at different times in three depth strata.

The results showed that time (day/night) had significant effects on the abundances of the small yellow croaker larvae and juveniles, which were significantly higher (*p* < 0.05) in the night (from 19:00 to 04:00 the next day) than in the daytime in every depth stratum (Table 1, Figure 6). The larvae and juveniles occurred in the bottom stratum during the entire sampling cycle, with their abundance varying periodically. In early May, the abundance peaked at 16:00 and 04:00 and reached low values at 10:00 and 22:00. In the other collection periods, abundance peaked at 22:00 and 07:00 and reached low values at 16:00 and 01:00 (Figure 6).

**Figure 7:**
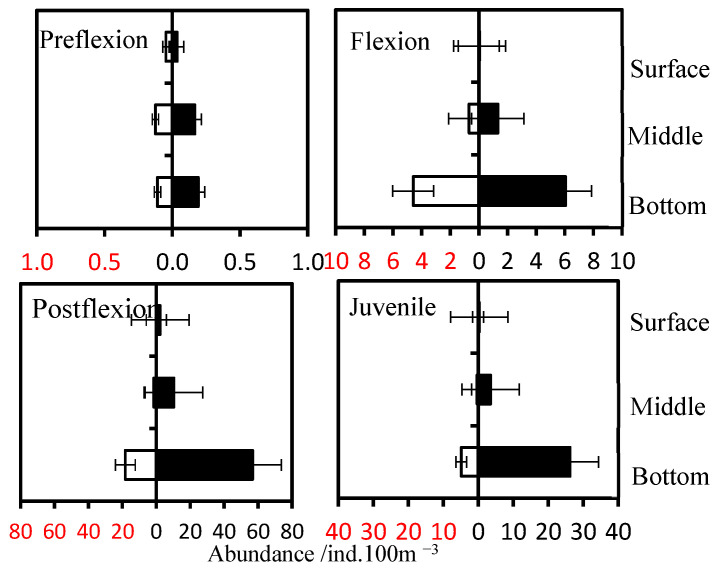
Mean vertical distribution of larvae and juveniles during the day (open bars) and night (filled bars).

The preflexion larvae were mainly localized in the middle and the bottom strata (Figure 7), while the latter development stages were mainly in the bottom layer. The abundance of preflexion larvae showed no difference between nighttime and daytime in each stratum (*p* > 0.05), while the flexion larvae migrated up to the middle stratum at night with higher abundance than that at daytime (*p* < 0.05), and the postflexion larvae and juveniles migrated up to the surface and middle strata at night with higher abundances than those recorded for the daytime (*p* < 0.05).

### 3.4. Relationship Between Abundance and Hydrographic Conditions

**Figure 8:**
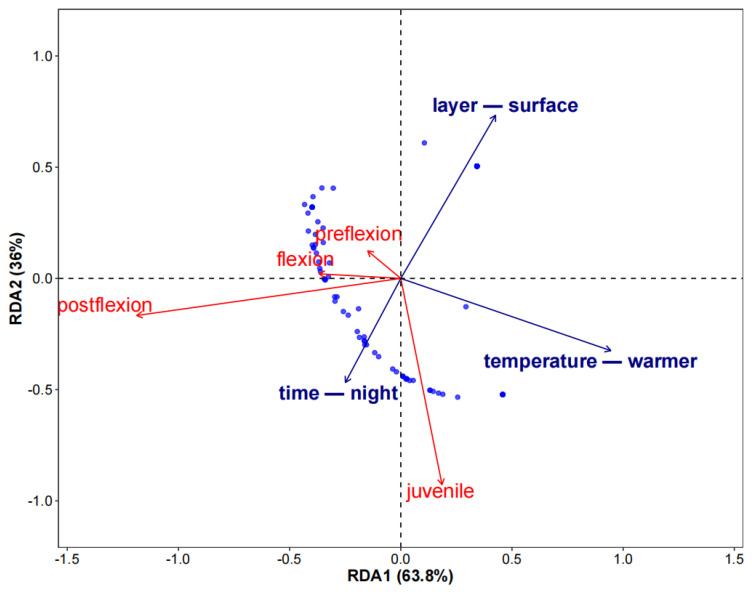
RDA biplot of fish abundance and environmental factors. The blue dots represent the sampling station. Three environmental variables are significant: temperature, layer and time.

The results of redundancy analysis (RDA) showed that environmental factors have significant constraint effects on the abundance of larvae and juveniles of the small yellow croaker (F = 22.86, *p* < 0.001). The model-adjusted coefficient of determination (R^2^adj = 0.408) showed that the three key environmental variables (water temperature, sampling water layer and sampling time) together explained 40.8% of the total variation. The eigenvalue decomposition showed that the cumulative interpretation rate of the first two axes was 99.8% (axis 1: 63.8%; axis 2: 36.0%), where axis 1 mainly represented the water temperature gradient and axis 2 reflected the circadian migration rhythm (Figure 8).

The response patterns of different developmental stages showed that postflexion larvae were concentrated in the negative area of axis 1, which was closely related to water temperature (r = −0.66), water layer (r = −0.38) and nocturnal sampling period (r = 0.24), showing low temperature response and light-avoiding sedimentation behavior. The juveniles were clustered in the negative area of axis 2, which was related to the water layer (r = −0.38) and temperature (r = 0.30), indicating that they preferred the bottom habitat. Compared with postflexion larvae and juveniles, the preflexion and flexion larvae had weaker selectivity to the water layer and time, and the individuals in the flexion stage also showed low temperature adaptability in the early stage of development (Figure 8).

**Figure 9:**
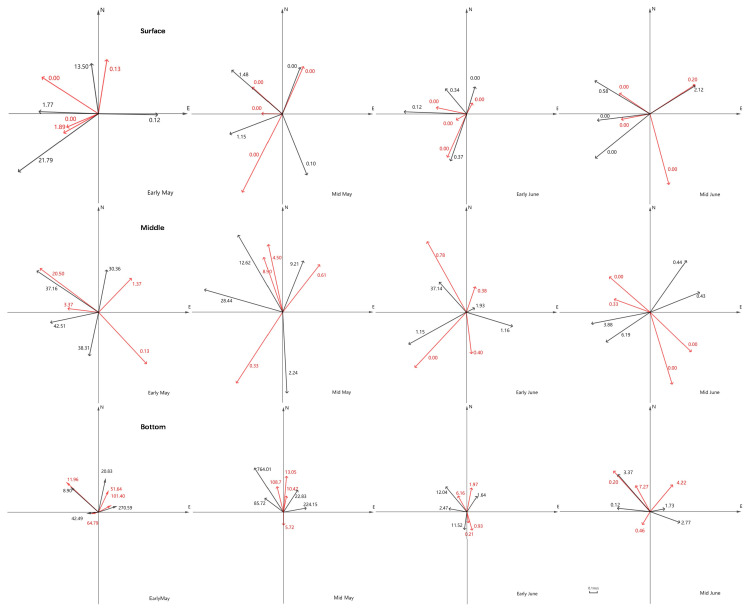
The abundance in each water layer at different current speeds and directions. Arrow direction and length: current direction and speed; numerical value: abundance (ind./100 m^3^); red arrow: daytime; black arrow: night.

Although the abundances of larvae and juveniles in the bottom and surface strata were greater at flood tide than at ebb tide, there was no significant difference between them. In contrast, the abundance in the middle layer was significantly higher at flood tide than that at ebb tide (*p* < 0.05), and it was more dominant at night than during the day. (Figure 9).

## 4. Discussion

### 4.1. Diel Vertical Migration

Fish larvae migration, being species-specific, may follow two patterns of diel vertical migration (DVM): DVM type I and DVM type II [15]. Larvae that follow DVM type I move upwards at night while larvae that follow DVM type II move upwards during the daytime. Larval concentrations examined off the Oregon coast suggested type-I DVM for *Stenobrachius leucopsarus* and *Sebastes* spp. larvae and type-II DVM for *Tarletonbeania crenularis* larvae [11]. *Sprattus sprattus* larvae migrated to the surface at night in the Baltic Sea [35], while the abundance of *Lateolabrax japonicus* significantly increased during the daytime in Ariake Bay [36]. It can be judged that the larval small yellow croaker DVM type I, but its migration phenomenon is not apparent. In contrast with species in which most individuals move toward the top of the water column during the night or descend toward deeper waters during the daytime [14], only a few larval and juvenile small yellow croaker individuals migrate upward at night.

DVM is often a size-related phenomenon, with the range of migration increasing with larval size [15]. It has been reported that the beginning of DVM requires a minimum species-specific size [37], which in some cases has been related to the change from endogenous to exogenous feeding [38], or to behavioral changes associated with ontogeny, e.g., the development of the caudal fin [37,39]. For the small yellow croaker, the DVM commences during the flexion stage and the migration range of postflexion larvae increases. This is probably related to increasing swimming ability. The insufficient swimming ability of preflexion larvae causes them to be unable to move toward the top of the water column under non-upwelling conditions [40]. The vertical distribution of preflexion larvae is related to adult spawning in the water column [14,41]. The small yellow croaker spawns over the slope at depths of about 20–50 m [18], and thus only a few of the preflexion larvae can distribute in the surface layer. During the flexion and postflexion stages, the swimming pattern changes from a burst and stop mode to a more continuous gliding mode or complex behaviors [40], so the flexion and postflexion small yellow croaker larvae gain the ability to migrate between different water strata.

As noted above, DVM has been well documented for the larvae of many marine fish species. A variety of theories have been put forth to explain DVM in larval fish, including predator avoidance [42], the pursuit of zooplankton prey [43], facilitated larval transport in varying tidal currents [44], optimization of the energetic advantage gained by larvae at certain depths in thermally stratified water [15] and the pursuit of optimum light conditions for larval survival [45]. The DVM strategy of the larval small yellow croaker is related to tidal current transport, which enables the larvae to select favorable conditions or avoid unfavorable ones [9,46]. Of course, there may be more factors for the diel vertical migration of the small yellow croaker, and this needs further study.

The characteristics of the vertical distribution of larval and juvenile small yellow croakers should be taken into account in fieldwork, because diurnal and ontogenetic changes in vertical distribution can have a strong influence on the interpretation of results from ichthyoplankton surveys [15].

### 4.2. Transport Pattern

Since before the 1980s, the YRE has been an important spawning and nursery ground for the small yellow croaker, but recent research has suggested that it is more responsible for the function of a nursery for the species [17,24,47]. There are three large spawning grounds around the YRE [19,48]: the Lüsi spawning ground, the Zhejiang coastal spawning ground and the outer YRE spawning ground (Figure 1). The small yellow croaker spawns from late April to mid-May in the Lüsi spawning ground [19], and it takes 36 days to develop from the egg to the postflexion larva [49]. Meanwhile, the small yellow croaker spawns during March and April in the spawning grounds of Zhejiang coast and outer YRE [19,48], with the egg needing about 46 days to develop into the postflexion larva [49]. Therefore, it is possible to infer that the larvae and juveniles distributed in the YRE originate from the Zhejiang coast spawning ground or the outer YRE spawning ground.

There are many complex flow features in the YRE, with net flow to the sea, which makes it particularly difficult for the larval small yellow croaker to be transported to the estuary. One possible behavioral mechanism leading to the up-estuary movement of the small yellow croaker larvae is selective tidal stream transport (STST) [50], in which larvae are up in the water column during rising tides and low in the water column during falling tides. STST has been demonstrated for many species of fish [3,4,5,51]. The preflexion larvae of the small yellow croaker, possibly with yolk-sac larvae and eggs, drift passively in ocean currents. At the beginning of the flexion stage, the larvae change their located depths using vertical migration, moving up to the water strata in which the current speed is faster during the flood tides and moving down to the deeper water in which the current speed is slower during the ebb tides. Depending on this pattern, the larvae of the small yellow croaker could ingress into the YRE nursery ground.

The selective tidal stream transport observed here (larvae ascending during flood tides) shares parallels with larval retention strategies in estuarine systems globally. The upstream migration and abundance of naked goby *Gobiosoma bosci* larvae in the Patuxent River indicate their utilization of tidal movements and rapid growth [52]. An investigation of *Anchoa mitchilli* larvae in the Hudson River estuary revealed complex migration behaviors influenced by tidal cycles and environmental gradients, supporting hypotheses of upstream larval transport [5]. The “retention–export balance” framework describes how reef fish larvae modulate vertical migration to either retain proximity to natal reefs or disperse to new habitats, depending on tidal and current regimes [6]. In the YRE, the retention–export balance is particularly critical due to the estuary’s high turbidity, strong tidal asymmetry and seasonal freshwater discharge. The observed STST behavior of the small yellow croaker larvae counteracts the seaward push of ebb-dominated flows, ensuring that larvae remain within the productive estuarine transition zone where salinity gradients and prey concentrations support growth.

Vertical migration consistent with STST may be triggered by pertinent stimuli. The changes in the depths at which fish larvae are located are probably regulated by endogenous rhythm, but appear to be triggered by environmental factors such as hydrographic conditions, light levels and turbulence [41]. The appropriate rhythm would have tidal periodicity synchronized with ambient tides and result in larvae being higher in the upper water column during the time of flood tides and in the lower water column during ebb tides [3]. There are some inadequacies in the STST process for the small yellow croaker, including, for example, the distribution of the larvae around the YRE, the environmental stimuli triggered and the detailed changes of currents in different water columns. All of these need further study.

## 5. Conclusions

In this paper, through four sampling cycles in the Yangtze River estuary during May and June 2015, the diel vertical migration and transport pattern of the larvae and juveniles of the small yellow croaker (*Larimichthys polyactis*) were studied. The abundance of the small yellow croaker larvae and juveniles was highest in the bottom stratum and lowest at the surface during all sampling cycles. Larvae at different developmental stages exhibited varying vertical distribution patterns, with postflexion larvae and juveniles showing significant diel vertical migration, moving up to the surface and middle strata at night. Environmental factors, including water temperature, sampling water layer and sampling time, significantly influenced the abundance and vertical distribution of the larvae and juveniles. The faster current speeds in the middle and surface strata during flood tides facilitated the up-estuary movement of the larvae. The larvae utilized selective tidal stream transport, moving up in the water column during flood tides and down during ebb tides to ingress into the nursery ground. The results preliminarily reveal the inshore migration strategy of the small yellow croaker in the Yangtze River estuary, and the characteristics of vertical distribution of the larval and juvenile small yellow croaker should be taken into account in future fieldwork.

## Figures and Tables

**Table 1:** Tests of between-subjects effects.

Source	Sum of Squares	df	Mean Square	F	Sig.	Partial Eta Squared
Corrected Model	34.72	23.00	1.51	10.76	0.00	0.77
Intercept	38.34	1.00	38.34	273.33	0.00	0.79
month	10.64	3.00	3.55	25.30	0.00	0.51
layer	13.85	2.00	6.92	49.37	0.00	0.58
day-night	3.60	1.00	3.60	25.66	0.00	0.26
month * layer	4.11	6.00	0.68	4.88	0.00	0.29
month * day-night	0.48	3.00	0.16	1.15	0.34	0.05
layer * day-night	0.64	2.00	0.32	2.27	0.11	0.06
Error	1.40	6.00	0.23	1.66	0.14	0.12
Total	10.10	72.00	0.14			
Corrected Total	83.15	96.00				

**Table 2:** Multiple comparisons with the Tukey HSD test.

(I) Month	(J) Month	Mean Difference (I − J)	Std. Error	Sig.	95% Confidence Interval	
					Lower Bound	Upper Bound
Early May	Mid-May	0.20	0.11	0.27	−0.09	0.48
	Early June	0.70	0.11	0.00	0.42	0.99
	Mid-June	0.79	0.11	0.00	0.51	1.07
Mid-May	Early May	−0.20	0.11	0.27	−0.48	0.09
	Early June	0.51	0.11	0.00	0.22	0.79
	Mid-June	0.59	0.11	0.00	0.31	0.88
Early June	Early May	−0.70	0.11	0.00	−0.99	−0.42
	Mid-May	−0.51	0.11	0.00	−0.79	−0.22
	Mid-June	0.09	0.11	0.85	−0.20	0.37
Mid-June	Early May	−0.79	0.11	0.00	−1.07	−0.51
	Mid-May	−0.59	0.11	0.00	−0.88	−0.31
	Early June	−0.09	0.11	0.85	−0.37	0.20
**(I) layer**	**(J) layer**					
Surface	Middle	−0.46	0.09	0.00	−0.69	−0.24
	Bottom	−0.93	0.09	0.00	−1.15	−0.71
Middle	Surface	0.46	0.09	0.00	0.24	0.69
	Bottom	−0.47	0.09	0.00	−0.69	−0.25
Bottom	Surface	0.93	0.09	0.00	0.71	1.15
	Middle	0.47	0.09	0.00	0.25	0.69

## Data Availability

The original contributions presented in this study are included in the article. Further inquiries can be directed to the corresponding author(s).
